# Genome-driven evaluation and redesign of PCR tools for improving the detection of virulence-associated genes in aeromonads

**DOI:** 10.1371/journal.pone.0201428

**Published:** 2018-08-15

**Authors:** Emilie Talagrand-Reboul, Fadua Latif-Eugenín, Roxana Beaz-Hidalgo, Sophie Colston, Maria-Jose Figueras, Joerg Graf, Estelle Jumas-Bilak, Brigitte Lamy

**Affiliations:** 1 Équipe Pathogènes Hydriques Santé Environnements, UMR 5569 HSM, Université de Montpellier, Montpellier, France; 2 Laboratoire de Bactériologie, Hôpitaux universitaires de Strasbourg, Strasbourg, France; 3 Unidad de Microbiología, Departamento de Ciencias Médicas Básicas, Facultad de Medicina y Ciencias de la Salud, IISPV, Universidad Rovira i Virgili, Reus, Spain; 4 Department of Molecular and Cell Biology, University of Connecticut, Storrs, Connecticut, United States of America; 5 Département d’Hygiène Hospitalière, CHRU de Montpellier, Montpellier, France; 6 Département de Bactériologie, CHU de Nice, Nice, France; Universidad Nacional de la Plata, ARGENTINA

## Abstract

Many virulence factors have been described for opportunistic pathogens within the genus *Aeromonas*. Polymerase Chain Reactions (PCRs) are commonly used in population studies of aeromonads to detect virulence-associated genes in order to better understand the epidemiology and emergence of *Aeromonas* from the environment to host, but their performances have never been thoroughly evaluated. We aimed to determine diagnostic sensitivity and specificity of PCR assays for the detection of virulence-associated genes in a collection of *Aeromonas* isolates representative for the genetic diversity in the genus. Thirty-nine *Aeromonas* strains belonging to 27 recognized species were screened by published PCR assays for virulence-associated genes (*act*, *aerA*, *aexT*, *alt*, *ascFG*, *ascV*, *ast*, *lafA*, *lip*, *ser*, *stx1*, *stx2A*). In parallel, homologues of the 12 putative virulence genes were searched from the genomes of the 39 strains. Of the 12 published PCR assays for virulence factors, the comparison of PCR results and genome analysis estimated diagnostic sensitivities ranging from 34% to 100% and diagnostic specificities ranged from 71% to 100% depending upon the gene. To improve the detection of virulence-associated genes in aeromonads, we have designed new primer pairs for *aerA/act*, *ser*, *lafA*, *ascFG* and *ascV*, which showed excellent diagnostic sensitivity and specificity. Altogether, the analysis of high quality genomic data, which are more and more easy to obtain, provides significant improvements in the genetic detection of virulence factors in bacterial strains.

## Introduction

*Aeromonas* are common inhabitants of aquatic environments and can be involved in fish and human diseases. They are frequently found in drinking water and food, including meat, fish, vegetables, and, more recently in ready-to-eat foods [1–6]. Among other illnesses, aeromonads are responsible for gastrointestinal syndromes following ingestion of contaminated food, and wound infection following water exposure in human [1–6], and for furunculosis and septicemia in fish causing major losses in the aquaculture sector [7].

The pathogenesis of *Aeromonas* infection is only partially elucidated [1,5,6,8] although a wide repertoire of virulence factors contributing to biofilm formation, cell adherence, invasion and cytotoxicity have been described [8]. It has been suggested that only certain subsets of strains within a species, referred as “pathotypes”, produce disease in certain individuals [1,9,10]; presumably due to differences in the content of virulence-associated genes. From this assumption, many studies leading to an abundant literature searched for virulent strains from water, fish, food or clinical samples using Polymerase Chain Reaction (PCR) for detecting virulence factors [11–14], notably for epidemiologic surveys (e. g., [15–17]). The accurate inference of virulence in such an approach depends on how accurate the PCR-based methods are for detecting genetic markers of virulence in aeromonads. But these methods have often been developed from a small number of strains, few species and for a specific purpose, and their performances have never been evaluated for the whole genus. Before raising conclusions on pathotype patterns in aeromonads, PCR performances at the genus level deserve to be questioned considering the high level of genetic polymorphism in the genus *Aeromonas* [18,19].

The recent availability of whole genome sequences (WGS) data from many *Aeromonas* isolates from various species [20,21] allows the search for virulence-associated genes by sequence comparison and the evaluation of performance of molecular methods. In the present study, we aimed to evaluate the accuracy of several widely used virulence PCRs by matching PCR results against available genomes. When necessary, accuracy was improved by the genome-driven design of new PCR tools.

## Materials and methods

### Bacterial strains and DNA extraction

Thirty-nine *Aeromonas* spp. strains were chosen to cover the whole genus. They belonged to 27 species (Table 1) among the 30 validated species in the *Aeromonas* genus at the time of January 2018. The current taxonomic affiliations are indicated for every strain with previously published names of strains indicated inside braces, where appropriate. Strains were grown on Trypticase Soy Agar at 35 °C for 16–24 h, and genomic DNA was extracted using the MasterPure^™^ DNA Purification Kit (Epicentre, USA).

**Table 1:** General features of the strains and genomes used in this study.

Strain (n = 39)	Source of isolation	Geno-me size (Mbp)	No of scaffolds	Average genome coverage	N50 (nt)	G+C content (%)	No of predicted CDSs	Level of assem-bly	Genome accession number	Reference
*A*. *allosacharophila* CECT 4199^T^	Infected eel	4.66	120	87	114,541	58.4	4,173	IHQ	PRJEB7019^a^	[20]
*A*. *australiensis* CECT 8023^T^	Irrigation water system	4.11	113	128	95,095	58.1	3,733	IHQ	PRJEB7021^a^	
*A*. *bestiarum* CECT 4227^T^	Fish	4.68	41	53	237,067	60.5	4,223	IHQ	PRJEB7022^a^	
*A*. *bivalvium* CECT 7113^T^	Cockles	4.28	69	30	149,050	62.3	3,909	IHQ	PRJEB7023^a^	
*A*. *caviae* CECT 838^T^	Guinea pig	4.47	111	95	101,663	61.6	4,081	IHQ	PRJEB7024^a^	
*A*. *dhakensis* CECT 7289^T^ {*A*. *aquariorum*}†	Aquaria of ornamental fish	4.69	78	117	163,504	61.7	4,266	IHQ	PRJEB7020^a^	
*A*. *dhakensis* CIP 107500 {*A*. *hydrophila* subsp. *dhakensis*}†	Human diarrheic stool	4.71	73	84	165,885	61.8	4,284	IHQ	PRJEB7048^a^	
*A*. *dhakensis* BVH28b	Human wound	4.89	68	130	150,860	61.7	4,466	IHQ	PRJEB9016^a^	[22]
*A*. *diversa* CECT 4254^T^	Human wound	4.06	37	116	203,531	61.5	3,711	IHQ	PRJEB7026^a^	[20]
*A*. *encheleia* CECT 4342^T^	Fish	4.47	35	112	380,984	61.9	4,076	IHQ	PRJEB7027^a^	
*A*. *enteropelogenes* CECT 4255^T^ {*A*. *trota*}†	Human stool	4.34	27	66	640,249	60.0	3,917	IHQ	PRJEB7043^a^	
*A*. *eucrenophila* CECT 4224^T^	Fresh water fish	4.54	22	50	441,212	61.1	4,113	IHQ	PRJEB7029^a^	
*A*. *fluvialis* LMG 24681^T^	River water	3.90	76	48	108,949	58.2	3,609	IHQ	PRJEB7030^a^	
*A*. *hydrophila* subsp. *hydrophila* CECT 839^T^	Tin of milk with fishy odor	4.74	1	-	4,744,448	61.5	4,119	C	CP000462^b^	[23]
*A*. *hydrophila* BVH25a	Human respiratory tract	5.10	130	44	84,371	60.9	4,598	IHQ	PRJEB9013^a^	[22]
*A*. *jandaei* CECT 4228^T^	Human stool	4.50	58	55	161,393	58.7	4,065	IHQ	PRJEB7031^a^	[20]
*A*. *media* CECT 4232^T^	River water	4.48	233	60	37,608	60.9	4,075	IHQ	PRJEB7032^a^	
*A*. *media* LMG 13464 {*A*. *caviae*}†	Infected fish	4.45	99	87	103,746	61.3	4,014	IHQ	PRJEB12347^a^	[21]
*A*. *media* CECT 7111	Oyster	4.41	92	70	108,504	61.6	3,998	IHQ	PRJEB12345^a^	
*A*. *molluscorum* CIP 108876^T^	Wedge-shells	4.23	309	9	21,565	59.2	3,946	IHQ	AQGQ01^b^	[24]
*Aeromonas* sp. genomospecies *paramedia* CECT 8838	Human diarrheic stool	4.46	128	99	78,349	62.2	4,086	IHQ	PRJEB12349^a^	[21]
*A*. *piscicola* LMG 24783^T^	Salmon	5.18	91	99	150,424	59.0	4,713	IHQ	PRJEB7033^a^	[20]
*A*. *popoffii* CIP 105493^T^	Drinking water production plant	4.76	105	67	113,495	58.4	4,331	IHQ	PRJEB7034^a^	
*A*. *rivipollensis* LMG 13459 {*A*. *caviae*}†	Infected fish	4.49	111	76	107,760	61.7	4,091	IHQ	PRJEB12346^a^	[21]
*A*. *rivipollensis* 76c {*A*. *media*}†	Human diarrheic stool	4.69	137	79	93,768	61.3	4,255	IHQ	PRJEB8966^a^	[22]
*A*. *rivipollensis* BVH40 {*A*. *media*}	Human stool	4.70	123	79	105,841	61.4	4,204	IHQ	PRJEB9017^a^	
*A*. *rivuli* DSM 22539^T^	Freshwater	4.53	102	99	155,151	60.0	4,149	IHQ	PRJEB7035^a^	[20]
*A*. *salmonicida* subsp. *salmonicida* CIP 103209^T^	Salmon	4.74	128	117	89, 543	58.5	4,442	IHQ	PRJEB7036^a^	
*A*. *sanarellii* LMG 24682^T^	Human wound	4.19	98	121	82,664	63.1	3,828	IHQ	PRJEB7037^a^	
*A*. *schubertii* CECT 4240^T^	Human wound	4.13	111	260	108,810	61.7	3,808	IHQ	PRJEB7038^a^	
*A*. *simiae* CIP 107798^T^	Healthy monkey	3.99	100	86	73,112	61.1	3,654	IHQ	PRJEB7039^a^	
*A*. *sobria* CECT 4245^T^	Fish	4.68	52	34	188,072	58.6	4,160	IHQ	PRJEB7040^a^	
*A*. *taiwanensis* LMG 24683^T^	Human wound	4.24	106	66	85,294	62.8	3,884	IHQ	PRJEB7041^a^	
*A*. *tecta* CECT 7082^T^	Human diarrheic stool	4.76	51	89	238,229	60.1	4,278	IHQ	PRJEB7042^a^	
*A*. *veronii* bv. *veronii* CECT 4257^T^	Human respiratory tract	4.52	52	59	181,171	58.8	4,070	IHQ	PRJEB7044^a^	
*A*. *veronii* bv. *sobria* LMG 13067	Environment	4.74	72	46	147,470	58.3	4,171	IHQ	PRJEB7051^a^	
*A*. *veronii* BVH25b	Human respiratory tract	4.66	35	63	241,725	58.7	4,185	IHQ	PRJEB9014^a^	[22]
*A*. *veronii* BVH26b	Human wound	4.58	48	73	180,501	58.7	4,107	IHQ	PRJEB9015^a^	
*A*. *veronii* 77c	Human diarrheic stool	4.61	42	78	230,104	58.6	4,124	IHQ	PRJEB9012^a^	

^a^: Performed at the Microbial Analysis, Resources and Services (MARS) facility at the University of Connecticut (Storrs, USA)

^b^: Obtained from GenBank, National Center for Biotechnology Information

^T^: Type strain

^†^ Previously published names are indicated inside braces.

Abbreviations: IHQ, Improved high quality draft genome; C, complete genome.

### Genome sequences

Thirty-seven draft genomes of *Aeromonas* spp., sequenced during previous works [20–22], were provided at the Microbial Analysis, Resources and Services (MARS) facility at the University of Connecticut (Storrs, USA) (Table 1) with a high quality level of draft genome on the basis of the quality of the sequencing and assembling (e.g., number of average genomes coverage and number of scaffolds) and of the verification of the automated annotation (16 housekeeping genes and 47 ribosomal protein-coding genes checked in every genome). Two other genomes were downloaded from GenBank (Table 1). To check the taxonomical assignation of genomes employed, we performed a ML phylogenetic tree of all the genomes (n = 39) calculated by the program kSNP3.1 [25], a kmer based alignment free method (see S1 Fig).

### Genome analysis

Genomic contigs and circular genomes were annotated by using the RAST annotation server to identify RNAs and protein-coding genes [26]. The genomes were queried for genes encoding for a set of virulence factors described in *Aeromonas* spp. by using reference protein sequences (Table 2), either using translated sequences of the validated subset of UniProtKB-SwissProt or annotated genes in UniprotKB-TrEMBL database. Sequence comparisons with reference protein sequences were performed with SEEDviewer that uses bidirectional protein-protein BLAST (BlastP) sequence comparison of translated open reading frames. Proteins with amino acid sequence similarities ≥65% and E-value ≤10^−10^ were considered to be homologous [27].

**Table 2:** Virulence-associated genes detected by genome analysis.

Strain (n = 39)	*aerA/act*	*ser*	*ast*	*alt/pla*	*stx1a*, *stx2a*	*aexT*	*aexU*	*ascFG*‡	*ascV*	*lafA*
	P09167^S^ (AerA) Q44063^E^ (Act)	A4SNU7^E^ (Ahe2) P31339^S^ (AspA) Q9RG23^E^ (Ahe2)	Q8VRN3^E^	Q44061^E^ (Alt) O87651^E^ (Pla)	E2DQN2^E^ (Stx1a) E2DQN6^E^ (Stx2a)	Q93Q17^S^	D5LUP3^E^	Q6WG33^E^ (AscF) Q6WG32^E^ (AscG)	A4SUH2^E^	Q93TL9^E^
*A*. *allosacharophila* CECT 4199^T^	-	+	-	+	-	-	+	-	-	-
*A*. *australiensis* CECT 8023^T^	+	+	-	+	-	-	-	-	-	-
*A*. *bestiarum* CECT 4227^T^	+	+	+	+	-	-	-	-	-	-
*A*. *bivalvium* CECT 7113^T^	-	+	-	+	-	-	-	-	-	+
*A*. *caviae* CECT 838^T^	-	-	-	+	-	-	-	-	-	+
*A*. *dhakensis* CECT 7289^T^ {*A*. *aquariorum*}†	+	+	-	+	-	-	-	-	-	-
*A*. *dhakensis* CIP 107500 {*A*. *hydrophila* subsp. *dhakensis*}†	+	+	-	+	-	-	+	+	+	+
*A*. *dhakensis* BVH28b	+	+	-	+	-	-	+	+	+	+
*A*. *diversa* CECT 4254^T^	+	-	-	+	-	-	-	+	+	+
*A*. *encheleia* CECT 4342^T^	-	+	-	+	-	-	+	+	+	+
*A*. *enteropelogenes* CECT 4255^T^ {*A*. *trota*}	+	+	+	+	-	-	-	-	-	+
*A*. *eucrenophila* CECT 4224^T^	+	+	-	+	-	-	-	-	-	+
*A*. *fluvialis* LMG 24681^T^	-	-	-	-	-	-	-	-	-	-
*A*. *hydrophila* subsp. *hydrophila* CECT 839^T^	+	+	+	+	-	-	-	-	-	-
*A*. *hydrophila* BVH25a	-	+	+	+	-	-	+	+	+	+
*A*. *jandaei* CECT 4228^T^	+	+	-	+	-	-	-	+	+	+
*A*. *media* CECT 4232^T^	-	-	-	+	-	-	-	-	-	-
*A*. *media* LMG 13464 {*A*. *caviae*}†	-	-	-	+	-	-	-	-	-	-
*A*. *media* CECT 7111	-	-	-	+	-	-	-	-	-	-
*A*. *molluscorum* CIP 108876^T^	+	-	-	+	-	-	-	-	-	-
*Aeromonas* sp. genomospecies *paramedia* CECT 8838	-	+	-	+	-	-	-	-	-	-
*A*. *piscicola* LMG 24783^T^	+	+	+	+	-	-	+	+	+	+
*A*. *popoffii* CIP 105493^T^	+	+	-	+	-	-	-	-	-	-
*A*. *rivipollensis* LMG 13459 {*A*. *caviae*}†	-	+	-	+	-	-	-	-	-	+
*A*. *rivipollensis* 76c {*A*. *media*}†	-	+	-	+	-	-	-	-	-	-
*A*. *rivipollensis* BVH40 {*A*. *media*}†	-	+	-	+	-	-	-	-	-	+
*A*. *rivuli* DSM 22539^T^	-	-	-	+	-	-	-	-	-	+
*A*. *salmonicida* subsp. *salmonicida* CIP 103209^T^	+	+	-	+	-	+	-	-	-	+
*A*. *sanarellii* LMG 24682^T^	-	-	-	+	-	-	-	-	-	-
*A*. *schubertii* CECT 4240^T^	+	-	-	+	-	-	-	+	+	+
*A*. *simiae* CIP 107798^T^	-	-	-	+	-	-	-	-	-	+
*A*. *sobria* CECT 4245^T^	+	+	+	+	-	-	-	-	-	-
*A*. *taiwanensis* LMG 24683^T^	-	-	-	+	-	-	-	-	-	+
*A*. *tecta* CECT 7082^T^	+	+	-	+	-	-	-	+	+	+
*A*. *veronii* bv. *veronii* CECT 4257^T^	+	+	-	+	-	-	-	-	-	-
*A*. *veronii* bv. *sobria* LMG 13067	+	+	-	+	-	-	-	-	-	-
*A*. *veronii* BVH25b	+	+	-	+	-	-	-	-	-	-
*A*. *veronii* BVH26b	+	+	-	+	-	-	+	+	+	+
*A*. *veronii* 77c	+	+	-	+	-	+	+	+	+	+

^S/E^: Accession numbers correspond to protein sequences in Swiss-prot (S) or TrEMBL databases (E).

^T^: Type strain

^†^ Previously published names are indicated inside braces.

^‡^ *ascF* and *ascG* are flanking genes.

### Polymerase Chain Reaction (PCR)

Both selected and new primers were used to amplify DNA from aerolysin/enterotoxin cytotoxic (*aerA*/*act*), heat-stable cytotonic enterotoxin (*ast*), heat-labile cytotonic enterotoxin (*alt*), lipase (*lip*), serine protease (*ser*), ADP-ribosylating toxins (*aexT* and *aexU*), shiga toxins 1 (*stx1*) and 2 (*stx2a*), T3SS needle proteins (*ascFG*), T3SS inner membrane channel protein (*ascV*) and lateral flagellin A (*lafA*) genes. PCR assays were carried out in a 25 μL reaction mixture containing 0.2 μM of each primer (Sigma-Aldrich, Saint-Louis, US), 0.2 mM of each deoxynucleoside triphosphate (dNTP) (Euromedex, Souffelweyersheim, France), 2.5 mM MgCl2 (Promega, Madison, US), and 1.25 U of GoTaq DNA polymerase (Promega, Madison, WI) in the appropriate reaction buffer containing 1.5 mM MgCl2 and 25 ng of genomic DNA as the template. The amplification conditions performed on a GeneAmp PCR System 9700 (Applied Biosystems, Foster city, US) were as follows: initial denaturation for 3 min at 95°C, followed by (1) main amplification program of the reference source specified in the Table 3 for already published PCR, with length of extension adapted to the efficacy of the DNA polymerase used (1 min/kb) when necessary, and a final extension step at 72°C for 10 min or (2) 32 amplification cycles as indicated in Table 4 for newly developed PCR. The PCR products and the 50 bp DNA ladder (New England BioLabs, Ipswich, US) were separated in 1.5% agarose gels in 0.5X TBE buffer-ethidium bromide 500 μg/mL and revealed under UV. Positive and negative controls were added in each PCR to assess the validity and specificity of amplification reaction. The positive controls of amplification corresponded to genomic DNA of one of the strains included in the study and for which the presence of gene of interest was checked on the basis of genomic analysis as follows: *A*. *veronii* 77C (*aer*/*act*, *aexT* and *ser*), *A*. *dhakensis* CIP 107500^T^ (*alt*, *ascFG*, *ascV* and *lafA*), *A*. *hydrophila* BVH 25a (*ast*). Specificity of the assays was confirmed using PCR-grade water and DNA from *A*. *fluvialis* LMG 24681^T^ which harbors in its genome none of the genes of interest. All strains were tested for all primer sets. For all the PCR assays and for each strain, the results of absence/presence of amplification was determined by two different readers and in two independent experiments on the basis of the expected size of amplified products and of the positive control.

**Table 3:** Performance of several PCRs used for the detection of virulence-associated genes in the genus *Aeromonas*.

Gene	Virulence factor	Prevalence estimated from WGS analysis (n = 39)	PCRRef.	Primers (nt.)	No of sequences in align-ment	No of primer mis-matches† (min-max)	DiagnosticSe % (CI95%)	Comments on primers / diagnostic sensitivity relationship	DiagnosticSp (%) (CI95%)	Comments on primers / specificity relationship
*aerA/ act*	Aerolysin AerA/ Cytotoxic enterotoxin Act	56% (n = 22)	[15]	AHCF1 (22) AHCR1 (22)	32 32	0–6 0–6	64 (41;83)	High variability in hybridization sequences of forward and reverse primers	100 (80;100)	-
			[30]	aer-f (20) aer-r (20)	32 32	0–4 0–2	91 (71;99)	-	82 (57;96)	aer-r: high GC content and self-end dimers
			This study	aer-1FX (20) aer-2R (19)	32 32	0–1 0–2	100 (85;100)	aer-1FX: degenerate nucleotides in the forward primer aer-f	94 (71;100)	Degenerate bases did not decrease specificity aer-2R: identical to aer-r but one 5’ end base deleted
*ser*	Serine protease	69% (n = 27)	[13]	Serine-f (20) Serine-r (20)	39 39	0–7 0–11	59 (39;78)	High variability in hybridization sequences of forward and reverse primers One nucleotide deleted in the reference sequences used to design the reverse primer (ENA X67043 and ENA AAF22245)	92 (62;100)	-
			This study	ser-1FX (21) ser-2RX (20)	39 39	0–2 0–0	96 (81;100)	Selection of less variable sequences Degenerate bases	92 (62;100)	Degenerate bases did not decrease specificity
*ast*	Cytotonic heat-stable enterotoxin	15% (n = 6)	[31]	ast-F (20) ast-R (20)	7 7	0–4 0–3	100 (54;100)	-	91 (76;98)	-
*alt*	cytotonic heat-labile enterotoxin	97% (n = 38)	[31]	alt-F (20) alt-R (19)	39 39	0–10 0–5	34 (20;51)	High variability in hybridization sequences of forward and reverse primers	-	Unevaluable specificity: insufficient number of strains for which *alt* was not detected in WGS (1/39)
			[13]	lip-f (18) lip-r (20)	39 39	0–1 0–2	68 (51;83)	-	-	
*stx1a*	Shiga toxin 1 subunit A	0% (n = 0)	[32]	Stx1-a (20) Stx1-b (20)	-	-	-	Not evaluable sensitivity: no strain for which *stx1a* or *stx2a* was detected in WGS	100 (82;100)	-
*stx2a*	Shiga toxin 2 subunit A	0% (n = 0)	[33]	S2Aup (19) S2Alp (19	-	-	-		100 (82;100)	-
*aexT*	ADP- ribosylating toxin AexT	5% (n = 2)	[34]	RASEXOS-L (18) RASEXOS-R (18)	9 9	0–1 0–1	2/2	-	100 (91;100)	-
*aexT* and/or *aexU*	ADP-ribosylating toxins	23% (n = 9)	This study	aexTU-1FX (19) aexTU-2RX (20)	21 21	0–2 0–2	100 (66;100)	-	100 (88;100)	-
*asc-FG*	T3SS needle proteins	28% (n = 11)	[16]	ascF-G-fwd (20) ascF-G-rev (20)	38 23	0–5 na	45 (17;77)	High variability in hybridization sequences of forward and reverse primers	74 (54;89)	ascF-G-fwd: high GC content, self-end dimers and 3’-end unstability
			This study	ascFG-1F (21) ascFG-2RX (20)	38 23	0–4 0–1	91 (59;100)	Selection of less variable sequences Degenerate bases	100 (88;100)	Degenerate bases did not decrease specificity
*asc-V*	T3SS inner membrane channel protein	28% (n = 11)	[16]	ascV-fwd (20) ascV-rev (20)	21 21	1–2 4–7	55 (23;83)	High variability in hybridization sequences of forward and reverse primers	71 (51;87)	ascV-fwd: high GC content
			This study	ascV-1F (19) ascV-2R (20)	21 21	0–1 0–2	100 (72;100)	Selection of less variable sequences	100 (88;100)	-
*lafA*	Lateral flagellin A	54% (n = 21)	[35]	Laf1 (18) Laf2 (17)	58 58	4–6 0–3	55 (34;80)	High variability in hybridization sequences of forward and reverse primers Wrong supplementary nucleotide (cytidine) in the 3’ region of laf1‡	100 (82;100)	-
			This study	lafA-1FX (19) lafA-2RX (18)	58 58	0–5 0–3	86 (64;97)	Selection of less variable sequences Degenerate bases Sensitivity <90%: high nucleotide variability throughout *lafA* sequence 55.8% of polymorphic positions‡	100 (81;100)	Degenerate bases did not decrease specificity

^†^Evaluated from the virulence-associated-gene/primers multiple alignments

^‡^ Evaluated from *lafA* multiple alignments

Abbreviations: CI95%, confidence interval 95%; na, not available; PCR, Polymerase Chain Reaction; Ref., References; WGS, Whole Genome Sequences.

**Table 4:** Primers designed in this study and amplification conditions of PCRs.

Virulence-associated gene	Primer sequences (5’-3’)	Amplified sequence length (bp)	Amplification conditions†		
			°C	Time (sec)	Number of cycles
*aerA* (syn: *act*)	aer-1FX: 5’-CCTAYRGYCTSAGCGAGAAG-3’ aer-2R: 5’-CAGTTCCAGTCCCACCACT-3’	430	94	60	32
			56	60	
			72	60	
*ser*	ser-1FX: 5’-GACAAYCGVGTSTTCAAAGAG-3’ ser-2RX: 5’-ACCACCARGTTCCAGAAGTT-3’	262	94	60	32
			59	60	
			72	30	
*aexT/aexU*	aexTU-1FX: 5’-TGGCVMTSAAAGAGTGGAT-3’ aexTU-2RX: 5’-GCARDGSRCCRTTGCCRGTC-3’	225	94	60	32
			63	60	
			72	45	
*asc-FG*	ascFG-1F: 5’-CAAGATCAACAAATGGTCGGT-3’ ascFG-2RX: 5-TTCAYCARRGADGASAGGCG-3’	262	94	60	32
			60	60	
			72	30	
*asc-V*	ascV-1F: 5’-CGCAAGGACATCATGCTGG-3’ ascV-2R: 5’-ATGATGATGAGGCCCGCGAT-3’	578	94	60	32
			65	60	
			72	45	
*lafA*	lafA-1FX: 5’-GATGYTGRGYACYGCCATG-3’ lafA-2RX: 5’-CATRTTGGARAGGTTRTTGAC-3’	619	94	60	32
			64	60	
			72	60	

Degenerate bases are underlined.

^**†**^The amplification conditions of PCR are preceded by an initial denaturation step at 95°C during 3 minutes and followed by a final elongation step at 72°C during 10 minutes.

### Primer design strategy

Nucleic sequences were aligned using the Clustal ω2 program within Seaview 4 package [28] (see S1 Files). Primers displaying a length of 18–21 nucleotides were designed from the conserved regions, and AmplifX 1.7.0 (CNRS, Aix-Marseille Université, France, http://crn2m.univ-mrs.fr/pub/amplifx-dist) was used to assess their intrinsic properties such as complexity (polyX and triplet repetitions), 3’ stability and self-dimer formation. The specificity of the new primers was checked with NCBI nucleotide-nucleotide BLAST.

### Challenging PCR accuracy characteristics

The accuracy of the PCR assays were evaluated by comparing the results of the PCR with those obtained by genome analysis, with WGS results considered as gold standard. Diagnostic sensitivity was defined as the percentage of ‘positive PCR results among all the strains for which the gene was detected in WGS’, while specificity was defined as the percentage of ‘negative PCR results among all the strains for which the gene was not detected in WGS’. Diagnostic sensitivity and specificity values below 90% were considered as insufficient. Exact confidence interval of a frequency, i.e., binomial confidence interval, was determined for each percentage, as described elsewhere [29] by using the web server http://statpages.info/confint.html.

## Results

### Virulence-associated gene selection

**Fig 1:**
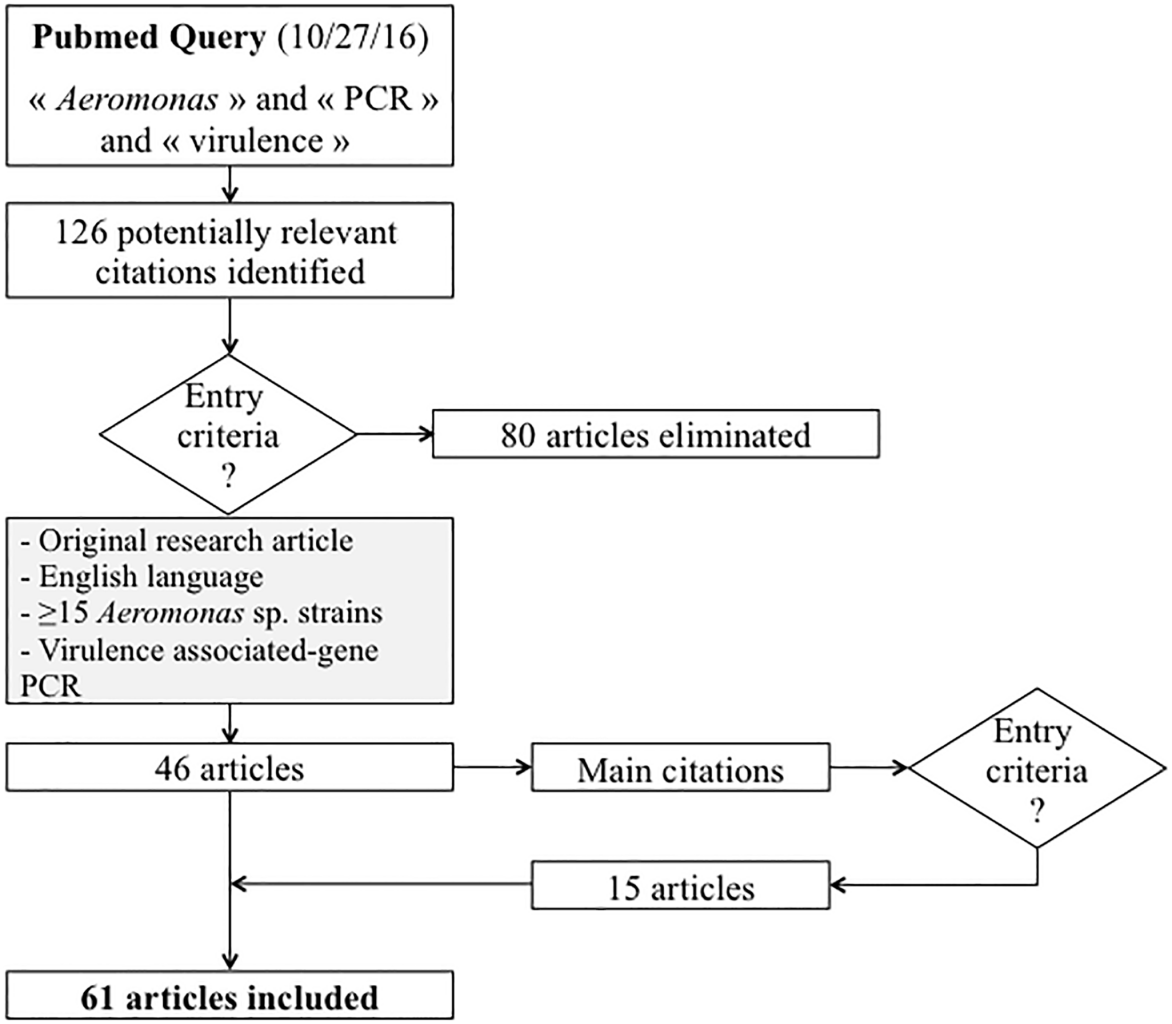
Flowchart for the literature analysis. The automatic Pubmed query using « *Aeromonas* », « PCR » and « virulence » as keywords generated 126 articles (October 27, 2016), among which 46 corresponded to the entry criteria. The reading of the relevant references quoted in these 46 selected articles led to the manual inclusion of 15 additional articles.

The literature analysis was used to inventory virulence genes commonly searched in *Aeromonas* and to identify the reference of the PCRs used in the different studies, as summarized in Fig 1. We selected 61 original research studies that performed virulence-associated gene PCRs for populations of at least 15 *Aeromonas* strains (Fig 1). From these 61 population studies, the origin of strains and distribution of *Aeromonas* species, the virulence-associated genes and the references of the PCRs method used are presented in the S1 Table. Among these 61 articles (see S1 Table for references), the most studied genes were *aerA* (n = 51), *alt* (n = 35), *act* (n = 30), *ast* (n = 30), *lip* (n = 19), *hlyA* (n = 17), *fla* (n = 15), *aexT* (n = 11), *aexU* (n = 2), *ascV* (n = 11), *ser* (n = 10), *gcat* (n = 9), *lipH3* (n = 8), *pla* (n = 8), *lafA* (n = 8), *ascFG* (n = 6), *exu* (n = 6), *ahyB* (n = 5), *stx1* (n = 5), *stx2* (n = 5) and *alp-1* (n = 5). These genes corresponded to different categories of virulence factors that are included in the pathotypes definition: hemolysins and related genes (*aerA*, *act*, *hlyA*), cytotonic enterotoxins genes (*alt*, *ast*), lipases genes (*lip*, *gcat*, *lipH3*, *pla*, *alp-1*), flagellar appendages genes (*fla*, *lafA*), type III secretion system effectors AexT and AexU genes (*aexT*, *aexU*), type III secretion system components genes (*ascV*, *ascFG*), serine protease gene (*ser*), nuclease gene (*exu*), elastase gene (*ahyB*) and shiga-toxins genes (*stx1*, *stx2*). We selected one or several genes from these virulence categories: *aerA* (aerolysin), *act* (cytolytic enterotoxin), *ast* (heat-stable cytotonic enterotoxin), *alt* (heat-labile cytotonic enterotoxin), *pla* (phospholipase A), *ser* (serine protease), *aexT* and aexU (ADP-ribosylating toxins), *stx1* and *stx2* (shiga-toxins), *ascFG* (type III secretion system needle proteins), *ascV* (type III secretion system inner membrane channel protein) and *lafA* (lateral flagellin A).

### Genome content in virulence-associated genes

We detected homologues for all the studied genes except for *stx1* and *stx2* (Table 2). The reference sequences of aerolysin gene *aerA* from *A*. *bestiarum Ah65* {considered in the original source as *A*. *hydrophila Ah65*} (SwissProt P09167) and Cytolytic enterotoxin gene *act* from *A*. *dhakensis* SSU {considered in the original source as *A*. *hydrophila* SSU} (UniprotKB-TrEMBL Q44063) shared a very high level of identity (95.1%) and similarity (97.2%) in protein sequence (493 amino-acids both). Similarly, the phospholipase A gene *pla* from *A*. *piscicola* AH-3 {considered in the original source as *A*. *hydrophila* AH-3} (UniprotKB-TrEMBL O87651) and the heat-labile cytotonic enterotoxin gene *alt* from *A*. *dhakensis* SSU (UniprotKB-TrEMBL Q44061) shared a very high level of identity (96.9%) and similarity (98.5%) in protein sequence, from an alignment of 89% query-cover of *alt* coding-sequence (368/385 amino-acids), *pla* coding-sequence spanning 805 amino-acids. These homology data and the presence of common flanking genes (data not shown), led to consider *aerA*/*act* and *alt*/*pla* as two single virulence-associated genes for all the strains included in the study. The *ser* gene that is putatively involved in the aerolysin activation by proteolytic cleavage was detected in 14 out of 17 *aerA*/*act* positive strains (Table 2). The genes coding for structural components of type III secretion system (T3SS) were detected simultaneously in the 11 T3SS gene positive strains (Table 2). The 248 residues from the N-terminal domain of the two ADP-ribosyltransferase toxins, AexT from *A*. *salmonicida* subsp. *salmonicida* ATCC 33658^T^ (SwissProt Q93Q17; 475 amino-acids) and AexU from *A*. *veronii* bv. *sobria* AeG1 (UniprotKB-TrEMBL D5LUP3, 512 amino-acids) presented a high level of identity (89.5%) and similarity (94.8%) in protein sequence, but they differed significantly in their C-terminal domain (< 20% similarity). All the strains that harbor the T3SS effector genes *aexT*/*aexU*, except *A*. *salmonicida* subsp. *salmonicida* CIP 103209^T^, also possessed the T3SS structural components. For the lateral flagellin gene, from 1 to 4 copies were detected in the genomes of the 21-*lafA* positive strains.

### PCR performance assessment

Results of the evaluation of PCR accuracy are presented in Table 3. Ability to amplify gene of interest was assessed for all genes but *stx1a* or *stx2a* because there were no strains for which the genes were detected in WGS (Table 2). The ability of the PCRs to amplify the gene of interest (i.e., diagnostic sensitivity, hereafter sensitivity) ranged from 34 to 100%. The PCR screening *aerA/act* that uses primers “*aer”*, and the PCRs screening the *ast* and *aexT* genes displayed high sensitivities with the tested set of strains, with values of 91%, 100% and 100%, respectively. However, the corresponding confidence intervals for *ast* and *aexT* PCR were large (range >40%) because the number of positive genomes was low, 6 and 2 out of the 39 genomes studied, respectively. On the opposite, with a sensitivity value of 64%, the PCR usually employed for screening *aerA/act* genes using primers “AHC” was disappointing and was associated with frequent false negative results. Similarly, low values of sensitivity were observed for the following genes: *ser* (59%), *alt/pla* (primers “*alt”*, 34%; primers “*lip”*, 68%), *ascFG* (45%), *ascV* (55%) and *lafA* (55%). Depending on the gene, the lack of sensitivity resulted from either an excessive variability in annealing of forward and/or reverse primers, or even errors in primer sequence transcript (e.g., Serine-r and Laf1; Table 3).

The specificities of the PCRs that could be evaluated ranged from 71% to 100% depending on the gene considered (Table 3). Specificity was not determined for *alt* because only one out of the 39 WGS lacked *alt* gene (*A*. *fluvialis* LMG 24681^T^). PCRs used for screening the following genes were highly specific: *aerA*/*act*-using primers “AHC” (100%), *ser* (92%), *ast* (91%), *aexT* (100%) and *lafA* (100%). The test method was insufficiently specific for *aerA*/*act* genes using primers “*aer*” (82%), *ascF-G* (74%) and *ascV* (71%) genes. Therefore, an overestimation of the prevalence of these virulence-associated factors by generation of false positive results was expected. Depending on the gene, the default of specificity likely resulted from PCR primers properties such as high GC content, self-end dimer formation and/or 3’-end unstability (Table 3).

### Genome driven design and evaluation of new PCR primers

Considering the low performance of some PCRs, we designed new primer pairs on the basis of multiple alignments (Table 4) with partial and full-length sequences retrieved from Genbank or EMBL databases (S2 Table), and from the WGS provided in this study (Table 1). To design the new primers, we selected low variable areas of sequence alignments and used degenerate bases in case of polymorphism. Newly designed primers were associated with an increased sensitivity of the virulence gene detection (Table 3). Meanwhile, the use of degenerate primers did not impair the specificity (Table 3). For screening of ADP-ribosylating toxins, we have designed primers matching in the homologous part shared by aexT and aexU genes that were associated with a sensitivity of 100%.

## Discussion

Many virulence factors are described in the genus *Aeromonas*, but to date, the pathogenicity of specific strains still cannot be predicted from the genome content. The genus *Aeromonas* is characterized by intraspecific and interspecific genetic diversities of both ribosomal and housekeeping genes that impair the definition of species and lead to the population structure of the genus in several complexes of species [19,36]. However, using the whole genome sequences and the average nucleotide identity index to compare them, all the species could be clearly delineated [20,21]. The huge variability observed in some housekeeping genes also occurs in virulence-associated genes, as already shown for the “S-layer protein” gene (*vapA*) that harbored variability up to 15% at the amino-acid level among the strains of different subspecies of *A*. *salmonicida* [18].

This study focuses on the performances of molecular techniques used for the detection of virulence-associated genes in *Aeromonas* strains. Some previous work aiming to detect virulence factors in the genus *Aeromonas* reported discrepancies between studies due to molecular methods used (e.g., [37]). The choice of strains used for the primer design was particularly questioned. The present study does not question the validity of previously published PCRs, especially when the original study aimed to screen the presence of virulence-associated genes in particular groups or species (e.g., in *A*. *caviae*, [38]), but aims to evaluate PCR performances at the genus level. The comparison of PCR results and high-quality WGS with automatic annotation and careful manual checking allows for the first time the proper evaluation of the PCR performances for detecting virulence-associated genes in aeromonads. Most new primers and PCR conditions proposed herein improve PCR performances although the design of degenerate primers was required for some virulence genes, i.e. when sequence polymorphisms are scattered all along the gene. Neither diagnostic sensitivity nor specificity was hampered in these cases.

Similar to most other population studies, our study suffers from some limitations. Studying whether strains are virulent may be challenging for opportunistic pathogens but at first, tools need to be accurate in establishing whether a gene is present or absent from genomes. Our study may have some potential bias in the strain sampling. But the collection of strains included in the study aimed to be as representative of the diversity found in the genus as possible, covering the interspecies diversity in the whole genus, given the studied genes are sought in a wide range of species [39], and the intra-species diversity of species that are the most frequently isolated in human clinical contexts [1,40]. Illustrating the unavoidable sampling bias, the shiga-toxin coding genes *stx* were not detected in this study by PCR nor by genome analysis. However, the *stx1* gene seems to be quite rare in the genus [41]. Another explanation would be *stx* instability due to the mobility of the *stx* phage that can lead to the gene loss after subculture [41] or may occur during storage before genome sequencing and PCR. The prevalence of some virulence markers was either too low (e.g., *stx*) or too high (e.g., *lip*, *alt*) so that PCR assay diagnostic sensitivity or specificity could not be evaluated, respectively. Despite the limitation in strain and sequence collections, this study provides a meaningful highlight in accuracy of widely used tools together with virulence gene content in a large collection of strains.

Besides performance assessment and method improvements, this study provides some interesting data on virulence in aeromonads, like the possible matching between several virulence markers identified in different *Aeromonas* species that could be established or confirmed. For instance, the homology between aerolysin gene *aerA* from *A*. *hydrophila* and cytotoxic enterotoxin gene *act* from *A*. *dhakensis* has previously been considered [42]. In fact, both toxins exhibit hemolytic, cytotoxic and enterotoxic activities and both cause lethality in mice [43–47]. Moreover, a similar mechanism of action, i.e., oligomerization followed by pore-formation, has been reported for these 2 toxins [46,48]. These two toxins are closely related but it is still possible that the aerolysin/cytotonic enterotoxin from *A*. *dhakensis* SSU possesses some structural and functional originality [46], and further characterization of the purified homologous proteins is required to clarify this point. The *aerA/act* PCR based on newly designed primers improved the diagnostic sensitivity for the panel of aeromonads covering the whole genus from 64–91% to 100%, and dramatically improved sensitivity for the species *A*. *dhakensis* (from 0–67% to 100%). Given the clinical importance of this species [49], an optimized performance for detecting the well-studied *aerA* virulence gene is critical, and should provide advances in the patho-epidemiological knowledge of the species. The putative correspondence between phospholipase A gene (*pla*) from *A*. *piscicola* AH-3 and heat-labile cytotonic enterotoxin gene (*alt*) from *A*. *dhakensis* SSU, was also observed by Balsalobre et al. [50]. In this case, the nomenclature of the virulence factor depends on the study [51,52]. The biological features are possibly divergent as mentioned by Merino et al. [52] and could depend on the allele of the gene *alt/pla* but this requires further studies.

Biological interpretation of the presence of virulence genes needs to be cautious because the presence of a virulence gene does not imply its expression in the host. In addition, we have to consider the pathogenic and non-pathogenic interactions depending on the context. For example, the aeromonads T3SS system is important for both pathogenicity and mutualism as demonstrated inside the leech microbiota [53]. In parallel, post-translational modifications for activation or effector translocation should be examined. The joint presence of the *ser* gene and *aer/act* should be considered because aerolysin is activated by a serine protease [54]. The *ser* gene was absent from the genomic data of three *aerA*/*act* positive strains included in this study: *A*. *diversa* CECT 4254^T^, *A*. *molluscorum* CIP 108876^T^ and *A*. *schubertii* CECT 4240^T^. Similarly, the presence of T3SS effectors AexT/U should be considered with respect to the presence of T3SS components, e.g., *ascFG* and *ascV*. Their presence is required for the delivery of AexT/U [55,56]. The presence of both genes *aexT* and *aexU* that we observed with the strains *A*. *veronii* 77C and BVH26b has already been mentioned for other *A*. *veronii* isolates and appears to be a frequent situation for strains carrying T3SS in the *A*. *veronii* group [37]. Therefore, for screening *aexT* and *aexU* genes in aeromonads, we propose an *aexT/U* PCR, followed by a molecular search of the *aexT* gene alone when the screening assay is positive.

In epidemiologic and pathogenesis studies of *Aeromonas*, large collections of strains were screened for their content in virulence-associated genes. Given the polymorphisms in virulence-associated genes highlighted in this study and because PCR assays to detect virulence associated genes were widely used without prior evaluation of their performance (diagnostic sensitivity and specificity) at the genus level (S1 Table), the published prevalence of virulence genes should be interpreted with caution. When the PCR performance is poor, the estimated prevalence is blurred with assay errors and does not reflect the true prevalence of the virulence factors. Importantly, assumptions on the pathogenic behavior of aeromonads have been made from virulence patterns obtained from defective/inaccurate tool results, and this may have led to wrong conclusions. This may contribute to the current complexity of virulence patterns in aeromonads, to the poor understanding of aeromonad virulence and to the frequent lack of links between the virulence factor pattern and the pathogenic behavior. Optimization of PCR conditions and primer is crucial to avoid biased data in applied and clinical microbiology, and in epidemiology.

The bacterial genomes, today widespread and easy to obtain, have a very high potential in numerous research areas, including the design of consolidated microbiological diagnostic tools, although they are maybe currently underused for this purpose. In our study, genomes allowed an accuracy evaluation of virulence-associated gene PCRs commonly used in the genus *Aeromonas* and their improvement by designing several improved oligonucleotide primers. WGS could obviously be used as primary data for searching virulence associated genes but WGS are still expensive when a large panel of strains is studied. Therefore, we propose here an accurate approach based on these genomic data to improve PCR methods aimed to detect virulence-associated genes in environmental bacteria.

Further advances in the knowledge of the genetic diversity and of the evolution of virulence-associated genes should improve the understanding of aeromonad adaptation to pathogenic behavior or patho-adaptation, and thereby the revision of pathotype definition.

## Supporting information

S1 Fig Genome-based ML phylogenetic tree. (DOCX)

S1 Files Genomic DNA and primer alignments. (ZIP)

S1 Table Literature survey and analysis. (DOCX)

S2 Table Additional nucleic sequence accession numbers used to design new primers. (DOCX)
