# Supplementary material for: Genome-driven evaluation and redesign of PCR tools for improving the detection of virulence-associated genes in aeromonads
Source: PLoS One. 2018 Aug 15;13(8):e0201428. doi: 10.1371/journal.pone.0201428 (PMC6093642; doi:10.1371/journal.pone.0201428)
Supplement: S1 Fig — (DOCX) [file pone.0201428.s001.docx]

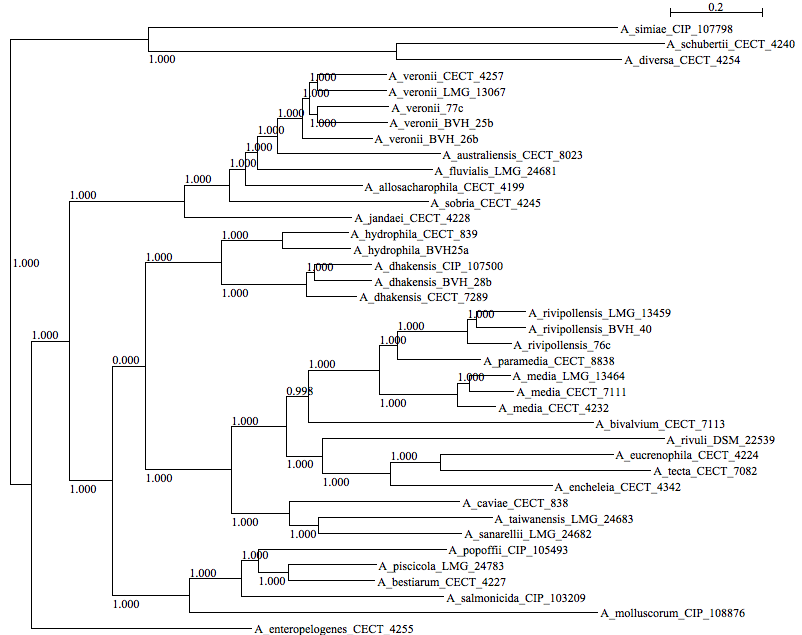


**S1 Figure. Genome-based ML phylogenetic tree.**

Phylogenetic reconstruction of assembled genome and related references was calculated using kSNP3.1, with k of 21 and maximum likelihood of a core single-nucleotide polymorphism (SNP) alignment. The scale bar indicates the number of substitutions per site.
