# Supplementary material for: Genome-driven evaluation and redesign of PCR tools for improving the detection of virulence-associated genes in aeromonads
Source: PLoS One. 2018 Aug 15;13(8):e0201428. doi: 10.1371/journal.pone.0201428 (PMC6093642; doi:10.1371/journal.pone.0201428)
Supplement: S1 Table — (DOCX) [file pone.0201428.s003.docx]

**S1 Table. Literature survey and analysis.**

| **Authors** | **Origin of strains** | **Virulence genes detected** | **References for the PCR used†** | **Distribution of *Aeromonas* species included in the study** |
| --- | --- | --- | --- | --- |
| Baloda et al., 1995 | Drinking water, fish, foods | *aerA* | 1 –specific test for *A. hydrophila* | *A. hydrophila* (n=60)  *A. sobria* (n=29) |
| Wang et al., 1996 | Human stools | *hem*/*aerA* | In-house PCR specific test for *A. caviae* | *A. caviae* (n=35) |
| Granum et al., 1998) | Food, water | *alt , aerA/act*, *aerA* promoter, | 2, in-house PCRs | *Aeromonas sp.* (n=31), |
| Kingombe et al., 1999 | Water, fish, environment, food, clinical samples | *aerA/act* | In-house PCR | *Aeromonas* sp. (n=350) |
| Heuzenroeder et al., 1999 | Water, environment, fish and clinical samples | *aerA, hlyA* | 3 | *A. hydrophila* (n=61), *A. veronii* bv. *sobria* (n=83), *A. caviae* (n=34) |
| Sechi et al., 2002 | Costal water, human stools, human blood | *aerA/act*, *tap*, *bfpA*, *bfpG* | 4, in-house PCRs | *A. hydrophila* (n=28), *A. sobria* (n=11), *A. salmonicida* (n=7) |
| Biscardi et al., 2002 | Mineral water, thermal water | *aerA* | 1 –specific test for *A. hydrophila* | *A. hydrophila* (n=18) |
| González-Rodríguez et al., 2002 | Fish | *aerA, hlyA* | 1, 3, 5 | *Aeromonas* sp. (n=35) |
| González-Serrano et al., 2002 | Fish, clinical sample | *aerA, hlyA* | 3, 5 | *A. hydrophila* (n=12), *A. veronii* (n=3) |
| Soler et al., 2002 | Freshwater and seawater | *aerA*, *lip*/*lipH3*/*pla*/*plc,* *gcat*, *ser*, *dns* | In-house PCRs | *A. popoffii* (n=26) |
| Castro-Escarpulli et al., 2003 | Frozen fish | *aerA*, *lip*/*lipH3*/*pla*/*plc,* *gcat*, *ser*, *dns* | 6 | *Aeromonas* sp. (n=82) |
| Abdullah et al., 2003 | Hospital environment, chicken carcasses and clinical samples | *aerA/act*, *alt, hlyA*, *opdA*, *hu-2* | 4, 7-9, in-house PCRs | *A. veronii* (n=34), *A. caviae* (n=13), *A. hydrophila* (n=5) |
| Wang et al., 2003 | Clinical samples | *ahh1*, *asa1*, *aerA* | In-house PCRs | *Aeromonas* sp. (n=121) |
| Chacón et al., 2003 | Clinical samples, food, fish, water | *aerA*, *lip*/*lipH3*/*pla*/*plc,* *gcat*, *ser*, *dns* | 6 | *Aeromonas* sp. (n=234) |
| Harf-Monteil et al., 2004 | Clinical samples | *hem*/*aerA* | 10 –specific test for *A. caviae* | *A. caviae* (n=28) |
| Sinha et al., 2004 | Human stools | *aerA*, *alt*, *ast*, *hlyA* | 1– species specific virulence test for *A. hydrophila*, 7, 9, 11 | *Aeromonas* sp. (n=164) |
| Bin Kingombe et al., 2004 | Food | *aerA/act* | 4 | *Aeromonas* sp. (n=34) |
| Sen and Rodgers, 2004 | Drinking water | *aerA/act, ahyB, lip*/*lipH3*/*pla*/*alp-1,* *fla*, *alt*, *ast* | 4, in-house PCRs | *Aeromonas* sp. (n=205) |
| Aguilera-Arreola et al., 2005 | Clinical samples, water, fish | *aerA*, *gcat*, *aspA*, *aexT*, *ascV*, *alt*, *ast*, *lafA* | 6, 12, in-house PCRs | *A. hydrophila* (n=31) |
| Nam and Joh, 2007 | Fish, water | *lafA, nuc, aerA*, *ser, gcat, lip* | In-house PCRs | *Aeromonas* sp. (n=444) |
| Aguilera-Arreola et al., 2007 | Clinical samples | *aerA*, *alt*, *ast*, *lafA* | 6, 13 | *A. hydrophila* (n=33), *A. caviae* (n=51), *A. veronii* (n=25) |
| Wu et al., 2007 | Clinical samples | *aerA/act, aerA, hlyA, alt*, *ast*, *ascV*, ascFG, *aexT* | 4, 12, 14-16 | *Aeromonas* sp. (n=116) |
| Chang et al., 2008 | Food | *act*, *alt*, *ast* | In-house PCRs | *Aeromonas* spp. (n=133) |
| Castilho et al., 2009 | Clinical samples, food, water | *hlyA*, *aerA*, *gidA*, *ast*, *alt* | 2, 7, 9 13, 17, 18 | *Aeromonas* spp. (n=117) |
| Silver and Graf, 2009 | Clinical samples, duck, eel, and leech | *ascFG*, *ascV*, *aexU*, *aexT* | 12, 19, in-house PCRs | *A. veronii* (n=20) |
| Balsalobre et al., 2009 | Water | *ast*, *alt*, *act/hlyA/aer* | 18, in-house PCRs | *A. hydrophila* (n=41), *A. jandaei* (n=46) |
| Pablos et al., 2009 | Drinking water | *aerA, hlyA, alt, ast, laf, stx*1, *stx*2 | 3, 5, 20, 21, in-house PCRs | *Aeromonas* sp. (n=35) |
| Pablos et al., 2010 | Human stools, drinking water | *aerA, hlyA, alt, ast, laf, stx*1, *stx*2, *asc-FG* | 3, 5, 12, 20-22 | *Aeromonas* sp. (n=44) |
| Alperi and Figueras, 2010 | Clinical samples | *stx*1, *stx*2 | 23 | *Aeromonas* spp. (n=80) |
| Nawaz et al., 2010 | Catfish | *aerA*, *act*, *ast*, *alt*, *lip*, *gcat*, *exu*, *ahyB*, *fla* | In-house PCRs | *A. veronii* (n=81) |
| Kingombe et al., 2010 | Food | *act*, *alt*, *ast* | In-house PCRs | *Aeromonas* spp. (n=537) |
| Khajanchi et al., 2010 | Water, human stools | *act*, *ast*, *alt*, *dam*, *tagA*, *gidA*, *eno*, *hlyA*, *ascV*, *aexU* | 2, 17, 24-30 | *Aeromonas* sp. (n=199) |
| Santos et al., 2011 | Lagoon water, food, human stools | *lafA*, *flaA* | 18, 20 | *A. caviae* (n=76) |
| Ottaviani et al., 2011 | Surface water, food, human stools | *act*, *aerA*, *alt,* *ast* | 31, 32 | *Aeromonas* sp. (n=142) |
| Li et al., 2011 | Fish, water | *aerA*, *alt*, *ahp* | In-house PCRs | *A. hydrophila* (n=83) |
| Figueras et al., 2011 | Chironomid egg masses | *ast*, *fla*, *alt*, *ahyB*, *pla*/ *lipH3*/ *alp-1*/ *lip*, *act*/ *aerA* /*hlyA*, *ascFG*, *aexT* | 4, 12, 18 | *A. aquariorum* (n=24), *A. hydrophila* (n=1), *A. veronii* (n=1) |
| Martino et al., 2011 | Fish, crustaceans, mollusks | *aexT*, *ascV*, *eno*, *asa1*, *ahh1*, *ast*, *act* | 4, 18, 33, in-house PCRs | *Aeromonas* sp. (n=77) |
| Aguilera-Arreola et al., 2011 | Human stools | *lafA*, *aer/hem*, *alt*, *ast*, *gcat* | In-house PCRs | *A. caviae* (n=25), *A. hydrophila* (n=18), *A. veronii* (n=11) |
| Berg et al., 2011 | Recreational water | *aerA*, *ahyB*, *pla*/*lip*/*lipH3* /*alp-1*, *fla*, *alt*, *ast* | 18 | *Aeromonas* sp. (n=176) |
| Yu and Chu, 2011 | Water | *aerA*, *ser*, *alt* | 34, in-house PCRs | *Aeromonas* sp. (n=18) |
| Nagar et al., 2011 | Food | *act*, *hly*, *aerA*, *ahyB*, *lip* | 3, 4, 5, 35 | *Aeromonas* sp. (n=22) |
| Puthucheary et al., 2012 | Clinical samples | *alt*, *ast*, *act*, *aerA*, *fla*, *lip*, *ela*, *ser*, *aexT*, *exu* | 24, 4, 36, 18, 19, 36-39 | *Aeromonas* sp. (n=94) |
| Hu et al., 2012 | Fish and water environment | *aerA*, *alt*, *act*, *ahp* | 18, 40 | *Aeromonas* sp. (n=202) |
| Lago et al., 2012 | Fish, water, food and mussels | *vapA*, *tap*, *fla*, *ascV*, *ascC*, *aexT*, *satA*, *aspA* | 6, 16, 18, 41-45 | *A. salmonicida* (n=42) |
| Carvalho et al., 2012 | Water | *lip*/*lipH3*/*pla*/*alp-1*, *aerA*/*act*, *ascV*, *aexT* | 4, 16, 18, 46 | *Aeromonas* sp. (n=80) |
| Ye et al., 2012 | Diseased turtles | *aerA, act, ast, alt* | 18, in-house PCR | *A. veronii* (n=15) |
| Igbinosa and Okoh, 2013 | Freshwater and wastewater treatment plant | *aerA*, *fla*, *lip*, *hlyA*, *alt*, *ast* | 39, 47 | *Aeromonas* sp. (n=45) |
| Yi et al., 2013 | Diseased eels | *act*, *alt*, *ast*, *gcat*, *exu*, *lip*, *fla* | 39 | *Aeromonas* sp. (n=70) |
| Skwor et al., 2014 | Lake water | *lafA, nuc, aerA*, *ser, gcat, lip* | 38 | *Aeromonas* sp. (n=119) |
| Aravena-Román et al., 2014 | Environmental and clinical samples | *aerA*/*hem*, *alt*, *lafA*, *flaA*, *aspA*, *vasH*, *ascV*, *aexT*, *bfpA*, *bfpG*, *stx1*, *stx2* | 6, 12, 13, 16, 18, 21, 48, 49 | *Aeromonas* sp. (n=129) |
| Robertson et al., 2014 | Water | *act*, *alt*, *ast*, *aerA* | 4, 33, 50 | *Aeromonas* sp. (n=15) |
| Jagoda et al., 2014 | Diseased fish | *aerA*, *act*, *ast*, *alt*, *fla*, *ser*, *exu* | 6, 39 | *Aeromonas* sp. (n=53) |
| Ghenghesh et al., 2014 | Food, water and clinical samples | *aerA*, *act*, *ast*, *alt* | 2, 17, 51, 52 | *Aeromonas* sp. (n=52) |
| Körkoca et al., 2014 | Animal and clinical samples | *aerA*, *hlyA*, *alt*, *ast*, *laf*, *ascFG*, *stx1*, *stx2* | 3, 5, 12, 20-22 | *Aeromonas* sp. (n=40) |
| Li et al., 2015 | Water, human stools | *aerA*, *act*, *ast*, *alt*, *hlyA*, *ahp*, *lip*, *fla*, *ascFG*, *laf* | 5, 12, 18, 22 | *Aeromonas* sp. (n=257) |
| Yano et al., 2015 | Shrimps | *aerA*, *act*, *alt*, *ast*, *ascV*, *aexT*, *fla*, *ela*, *lip* | 18, 37, 53, 54 | *Aeromonas* sp. (n=87) |
| Abu-Elala et al., 2015 | Chicken, fish | *aerA*, *act*, *lip*, *ser*, *cai* | 38, 55 | *A. sobria* (n=4), *A. hydrophila* (n=11), *A. caviae* (n=9) |
| Olaniran et al., 2015 | Wastewater, river water | *aerA*, *lip* | 56 | *Aeromonas* sp. (n=100) |
| Khor et al., 2015 | Lake water | *aerA*, *act*, *exu*, *ser*, *fla*, *eno*, *ela*, *aexT*, *aexU*, *lip*, *dam*, *alt*, *ast*, *ascV*, *hlyA* | 4, 13, 18, 25, 28-30, 36-39, 57 | *Aeromonas* sp. (n=102) |
| Albarral et al., 2016 | Clinical and environmental samples | *aerA*, *alt*, *ast*, *ascV* | 6, 13, 18, 56, in-house PCRs | *Aeromonas* sp. (n=127) |
| Králová et al., 2016 | Water, clinical samples | *aerA*/*act*, *ast*, *alt*, *ahh1*, *asa1*, *fla*, *lip*/*lipH3*/*pla*/*alp-1* | 4, 18, 33 | *Aeromonas* sp. (n=84) |

†Reference of the PCR used: 1: Pollard et al., 1990; 2: Chopra et al., 1993; 3: Wong et al., 1998; 4: Kingombe et al., 1999; 5: Santos et al. 1999; 6: Soler et al., 2002; 7: Heuzenroeder et al., 1999, 8: Ørmen et al., 2003; 9: Granum et al., 1998; 10: Wang et al., 1996; 11: Chopra et al., unpublished; 12: Chacón et al., 2004; 13: Aguilera-Arreola et al., 2005; 14: González-Serrano et al., 2002; 15: Albert et al., 2000; 16: Braun et al., 2002; 17:Sha et al., 2002; 18: Sen and Rodgers, 2004; 19: Vilches et al., 2004; 20: Gavín et al., 2003; 21: Paton and Paton, 1998; 22: Martínez et al., 2009; 23: Takara Biomedicals; 24: Chopra et al., 1996; 25: Erova et al., 2006; 26: Pillai et al., 2006; 27: Sha et al., 2004, 28: Sha et al., 2003, 29: Erova et al., 2007, 30: Sha et al., 2007; 31: Chang et al., 2008; 32: Kannan et al., 2001; 33: Wang et al., 2003; 34: Yu et al., 2008; 35: Sen, 2005; 36: Vilches et al., 2008; 37: Chacón et al., 2003; 38: Nam and Joh, 2007; 39: Nawaz et al., 2010; 40: Li et al., 2011; 41: Burr et al., 2005; 42: Ebanks et al., 2006; 43: Gustafson et al., 1992; 44: Madetoja et al., 2003; 45: Nilsson et al., 2006; 46: Wu et al., 2007; 47: Pablos et al., 2009; 48: Sechi et al., 2002; 49: Suarez et al., 2008; 50: Kingombe et al., 2010; 51: Anguita et al., 1993; 52: Song et al., 2004; 53: Aguilera-Arreola et al., 2007; 54: Martino et al., 2011; 55: Hu et al., 2012; 56: Igbinosa and Okoh, 2013; 57: Sha et al., 2005.

Abbreviations: *act*, cytolytic enterotoxin gene*; aerA*, aerolysin gene; *aexT*, ADP-ribosyltransferase toxin AexT gene; *aexU*, ADP-ribosyltransferase toxin AexU gene; *ahh1*, hemolysin Ahh1 gene; *ahp*, serine protease Ahp gene; *ahyB*, elastase gene; *alp-1*, lipase Alp-1 gene; *alt*, heat-labile cytotonic enterotoxin gene; *asa1*, hemolysin Asa1 gene*; ascC*, type III secretion system protein AscC gene; *ascFG*, type III secretion system protein AscF and AscG genes; *ascV*, inner membrane compound of type III secretion system gene; *aspA*, serine protease AspA gene; *ast*, heat-stable cytotonic enterotoxin gene; *bfpA* and *bfpG*, bundle forming pilus genes; *cai*, temperature sensitive protease Epr gene; *dam*; DNA adenine methyltransferase gene; *dns*, extracellular deoxyribonuclease gene; *ela*, elastase gene; *eno*, enolase gene; *exu*, extracellular deoxyribonuclease Exu gene; *fla*, polar flagellin gene; *hem*, hemolysin gene; *gcat*, glycerol-phospholipid-cholesterol-acetyltransferase gene; *gidA*, glucose-inhibited division protein gene; *hlya*, putative membrane protein insertion efficiency factor gene; *hu-2*, histone-like protein gene; *lafA*, lateral flagellin gene; *lip*, lipase gene; *lipH3*, lipase H3 gene; *nuc*, nuclease gene; *opdA*, oligopeptidase gene; *pla*, phospholipase A gene; *plc*, phospholipase C gene; *satA*, glycerol-phospholipid-cholesterol-acetyltransferase SatA gene; *ser*, serine protease gene; *stx1* and *stx2*, shiga-toxin genes; *tagA*, ToxR regulated lipoprotein gene; *tap*, type IV pilus gene; *vapA*, surface array protein gene; *vasH*, type VI secretion system component gene.

**References of the S1 Table.**

Abdullah, A. I., Hart, C. A., and Winstanley, C. (2003). Molecular characterization and distribution of virulence-associated genes amongst *Aeromonas* isolates from Libya. *J. Appl. Microbiol.* 95, 1001–1007.

Abu-Elala, N., Abdelsalam, M., Marouf, S., and Setta, A. (2015). Comparative analysis of virulence genes, antibiotic resistance and gyrB-based phylogeny of motile *Aeromonas* species isolates from Nile tilapia and domestic fowl. *Lett. Appl. Microbiol.* 61, 429–436. doi:10.1111/lam.12484.

Aguilera-Arreola, M. G., Hernández-Rodríguez, C., Zúñiga, G., Figueras, M. J., and Castro-Escarpulli, G. (2005). *Aeromonas hydrophila* clinical and environmental ecotypes as revealed by genetic diversity and virulence genes. *FEMS Microbiol. Lett.* 242, 231–240. doi:10.1016/j.femsle.2004.11.011.

Aguilera-Arreola, M. G., Hernández-Rodríguez, C., Zúñiga, G., Figueras, M. J., Garduño, R. A., and Castro-Escarpulli, G. (2007). Virulence potential and genetic diversity of *Aeromonas caviae*, *Aeromonas veronii*, and A*eromonas hydrophila* clinical isolates from Mexico and Spain: a comparative study. *Can. J. Microbiol.* 53, 877–887. doi:10.1139/W07-051.

Aguilera-Arreola, M. G., Martínez, A. A. C., and Castro-Escarpulli, G. (2011). An in-house multiplex pcr method to detect of putative virulence factors in *Aeromonas* species. *Braz. J. Microbiol. Publ. Braz. Soc. Microbiol.* 42, 1314–1320. doi:10.1590/S1517-838220110004000011.

Albarral, V., Sanglas, A., Palau, M., Miñana-Galbis, D., and Fusté, M. C. (2016). Potential pathogenicity of *Aeromonas hydrophila* complex strains isolated from clinical, food, and environmental sources. *Can. J. Microbiol.* 62, 296–306. doi:10.1139/cjm-2015-0466.

Albert, M. J., Ansaruzzaman, M., Talukder, K. A., Chopra, A. K., Kuhn, I., Rahman, M., et al. (2000). Prevalence of enterotoxin genes in *Aeromonas* spp. isolated from children with diarrhea, healthy controls, and the environment. *J. Clin. Microbiol.* 38, 3785–3790.

Alperi, A., and Figueras, M. J. (2010). Human isolates of *Aeromonas* possess Shiga toxin genes (*stx1* and *stx2*) highly similar to the most virulent gene variants of *Escherichia coli*. *Clin. Microbiol. Infect.* 16, 1563–1567. doi:10.1111/j.1469-0691.2010.03203.x.

Anguita, J., Rodríguez Aparicio, L. B., and Naharro, G. (1993). Purification, gene cloning, amino acid sequence analysis, and expression of an extracellular lipase from an *Aeromonas hydrophila* human isolate. *Appl. Environ. Microbiol.* 59, 2411–2417.

Aravena-Román, M., Inglis, T. J. J., Riley, T. V., and Chang, B. J. (2014). Distribution of 13 virulence genes among clinical and environmental *Aeromonas* spp. in Western Australia. *Eur. J. Clin. Microbiol. Infect. Dis. Off. Publ. Eur. Soc. Clin. Microbiol.* 33, 1889–1895. doi:10.1007/s10096-014-2157-0.

Baloda, S. B., Krovacek, K., Eriksson, L., Linné, T., and Månsson, I. (1995). Detection of aerolysin gene in *Aeromonas* strains isolated from drinking water, fish and foods by the polymerase chain reaction. *Comp. Immunol. Microbiol. Infect. Dis.* 18, 17–26.

Balsalobre, L. C., Dropa, M., Matté, G. R., and Matté, M. H. (2009). Molecular detection of enterotoxins in environmental strains of *Aeromonas hydrophila* and *Aeromonas jandaei*. *J. Water Health* 7, 685–691. doi:10.2166/wh.2009.082.

Berg, K. A., Lyra, C., Niemi, R. M., Heens, B., Hoppu, K., Erkomaa, K., et al. (2011). Virulence genes of *Aeromonas* isolates, bacterial endotoxins and cyanobacterial toxins from recreational water samples associated with human health symptoms. *J. Water Health* 9, 670–679. doi:10.2166/wh.2011.206.

Bin Kingombe, C. I., Huys, G., Howald, D., Luthi, E., Swings, J., and Jemmi, T. (2004). The usefulness of molecular techniques to assess the presence of *Aeromonas* spp. harboring virulence markers in foods. *Int. J. Food Microbiol.* 94, 113–121. doi:10.1016/S0168-1605(03)00105-3.

Biscardi, D., Castaldo, A., Gualillo, O., and de Fusco, R. (2002). The occurrence of cytotoxic *Aeromonas hydrophila* strains in Italian mineral and thermal waters. *Sci. Total Environ.* 292, 255–263.

Braun, M., Stuber, K., Schlatter, Y., Wahli, T., Kuhnert, P., and Frey, J. (2002). Characterization of an ADP-ribosyltransferase toxin (AexT) from *Aeromonas salmonicida* subsp. *salmonicida*. *J. Bacteriol.* 184, 1851–1858.

Burr, S. E., Pugovkin, D., Wahli, T., Segner, H., and Frey, J. (2005). Attenuated virulence of an *Aeromonas salmonicida* subsp. *salmonicida* type III secretion mutant in a rainbow trout model. *Microbiol. Read. Engl.* 151, 2111–2118. doi:10.1099/mic.0.27926-0.

Carvalho, M. J., Martínez-Murcia, A., Esteves, A. C., Correia, A., and Saavedra, M. J. (2012). Phylogenetic diversity, antibiotic resistance and virulence traits of *Aeromonas* spp. from untreated waters for human consumption. *Int. J. Food Microbiol.* 159, 230–239. doi:10.1016/j.ijfoodmicro.2012.09.008.

Castilho, M. C. B., Castro, T. L. A., Araújo, V. S., Trajano, R. S., Santos, P. A., Pimenta, P. M. C., et al. (2009). High frequency of hemolytic and cytotoxic activity in *Aeromonas* spp. isolated from clinical, food and environmental in Rio de Janeiro, Brazil. *Antonie Van Leeuwenhoek* 96, 53–61. doi:10.1007/s10482-009-9335-6.

Castro-Escarpulli, G., Figueras, M. J., Aguilera-Arreola, G., Soler, L., Fernández-Rendón, E., Aparicio, G. O., et al. (2003). Characterisation of *Aeromonas* spp. isolated from frozen fish intended for human consumption in Mexico. *Int. J. Food Microbiol.* 84, 41–49.

Chacón, M. R., Figueras, M. J., Castro-Escarpulli, G., Soler, L., and Guarro, J. (2003). Distribution of virulence genes in clinical and environmental isolates of *Aeromonas* spp. *Antonie Van Leeuwenhoek* 84, 269–278.

Chacón, M. R., Soler, L., Groisman, E. A., Guarro, J., and Figueras, M. J. (2004). Type III secretion system genes in clinical *Aeromonas* isolates. *J. Clin. Microbiol.* 42, 1285–1287.

Chang, Y.-C., Wang, J.-Y., Selvam, A., Kao, S.-C., Yang, S.-S., and Shih, D. Y.-C. (2008). Multiplex PCR detection of enterotoxin genes in *Aeromonas* spp. from suspect food samples in northern Taiwan. *J. Food Prot.* 71, 2094–2099.

Chopra, A. K., Houston, C. W., Peterson, J. W., and Jin, G. F. (1993). Cloning, expression, and sequence analysis of a cytolytic enterotoxin gene from *Aeromonas hydrophila*. *Can. J. Microbiol.* 39, 513–523.

Chopra, A. K., Peterson, J. W., Xu, X. J., Coppenhaver, D. H., and Houston, C. W. (1996). Molecular and biochemical characterization of a heat-labile cytotonic enterotoxin from *Aeromonas hydrophila*. *Microb. Pathog.* 21, 357–377. doi:10.1006/mpat.1996.0068.

Ebanks, R. O., Knickle, L. C., Goguen, M., Boyd, J. M., Pinto, D. M., Reith, M., et al. (2006). Expression of and secretion through the *Aeromonas salmonicida* type III secretion system. *Microbiol. Read. Engl.* 152, 1275–1286. doi:10.1099/mic.0.28485-0.

Erova, T. E., Pillai, L., Fadl, A. A., Sha, J., Wang, S., Galindo, C. L., et al. (2006). DNA adenine methyltransferase influences the virulence of *Aeromonas hydrophila*. *Infect. Immun.* 74, 410–424. doi:10.1128/IAI.74.1.410-424.2006.

Erova, T. E., Sha, J., Horneman, A. J., Borchardt, M. A., Khajanchi, B. K., Fadl, A. A., et al. (2007). Identification of a new hemolysin from diarrheal isolate SSU of *Aeromonas hydrophila*. *FEMS Microbiol. Lett.* 275, 301–311. doi:10.1111/j.1574-6968.2007.00895.x.

Figueras, M. J., Beaz-Hidalgo, R., Senderovich, Y., Laviad, S., and Halpern, M. (2011). Re-identification of *Aeromonas* isolates from chironomid egg masses as the potential pathogenic bacteria *Aeromonas aquariorum*. *Environ. Microbiol. Rep.* 3, 239–244. doi:10.1111/j.1758-2229.2010.00216.x.

Gavín, R., Merino, S., Altarriba, M., Canals, R., Shaw, J. G., and Tomás, J. M. (2003). Lateral flagella are required for increased cell adherence, invasion and biofilm formation by *Aeromonas* spp. *FEMS Microbiol. Lett.* 224, 77–83.

Ghenghesh, K. S., Ahmed, S. F., Cappuccinelli, P., and Klena, J. D. (2014). Genospecies and virulence factors of *Aeromonas* species in different sources in a North African country. *Libyan J. Med.* 9, 25497.

González-Rodríguez, M. N., Santos, J. A., Otero, A., and García-López, M. L. (2002). PCR detection of potentially pathogenic aeromonads in raw and cold-smoked freshwater fish. *J. Appl. Microbiol.* 93, 675–680.

González-Serrano, C. J., Santos, J. A., García-López, M. L., and Otero, A. (2002). Virulence markers in *Aeromonas hydrophila* and *Aeromonas veronii* biovar *sobria* isolates from freshwater fish and from a diarrhoea case. *J. Appl. Microbiol.* 93, 414–419.

Granum, P. E., O’Sullivan, K., Tomás, J. M., and Ormen, O. (1998). Possible virulence factors of *Aeromonas* spp. from food and water. *FEMS Immunol. Med. Microbiol.* 21, 131–137.

Gustafson, C. E., Thomas, C. J., and Trust, T. J. (1992). Detection of *Aeromonas salmonicida* from fish by using polymerase chain reaction amplification of the virulence surface array protein gene. *Appl. Environ. Microbiol.* 58, 3816–3825.

Harf-Monteil, C., Prévost, G., and Monteil, H. (2004). [Virulence factors of clinical *Aeromonas caviae* isolates]. *Pathol. Biol. (Paris)* 52, 21–25. doi:10.1016/j.patbio.2003.09.011.

Heuzenroeder, M. W., Wong, C. Y. F., and Flower, R. L. P. (1999). Distribution of two hemolytic toxin genes in clinical and environmental isolates of *Aeromonas* spp.: correlation with virulence in a suckling mouse model. *FEMS Microbiol. Lett.* 174, 131–136. doi:10.1111/j.1574-6968.1999.tb13559.x.

Hu, M., Wang, N., Pan, Z. H., Lu, C. P., and Liu, Y. J. (2012). Identity and virulence properties of *Aeromonas* isolates from diseased fish, healthy controls and water environment in China. *Lett. Appl. Microbiol.* 55, 224–233. doi:10.1111/j.1472-765X.2012.03281.x.

Igbinosa, I. H., and Okoh, A. I. (2013). Detection and distribution of putative virulence associated genes in *Aeromonas* species from freshwater and wastewater treatment plant. *J. Basic Microbiol.* 53, 895–901. doi:10.1002/jobm.201200351.

Jagoda, S. S. S. de S., Wijewardana, T. G., Arulkanthan, A., Igarashi, Y., Tan, E., Kinoshita, S., et al. (2014). Characterization and antimicrobial susceptibility of motile aeromonads isolated from freshwater ornamental fish showing signs of septicaemia. *Dis. Aquat. Organ.* 109, 127–137. doi:10.3354/dao02733.

Kannan, S., Suresh Kanna, P., Karkuzhali, K., Chattopadhyay, U. K., and Pal, D. (2001). Direct detection of diarrheagenic Aeromonas from faeces by polymerase chain reaction (PCR) targeting aerolysin toxin gene. *Eur. Rev. Med. Pharmacol. Sci.* 5, 91–94.

Khajanchi, B. K., Fadl, A. A., Borchardt, M. A., Berg, R. L., Horneman, A. J., Stemper, M. E., et al. (2010). Distribution of virulence factors and molecular fingerprinting of *Aeromonas* species isolates from water and clinical samples: suggestive evidence of water-to-human transmission. *Appl. Environ. Microbiol.* 76, 2313–2325. doi:10.1128/AEM.02535-09.

Khor, W. C., Puah, S. M., Tan, J. A. M. A., Puthucheary, S. D., and Chua, K. H. (2015). Phenotypic and genetic diversity of *Aeromonas* species isolated from fresh water lakes in Malaysia. *PloS One* 10, e0145933. doi:10.1371/journal.pone.0145933.

Kingombe, C. I. B., D’Aoust, J.-Y., Huys, G., Hofmann, L., Rao, M., and Kwan, J. (2010). Multiplex PCR Method for Detection of Three Aeromonas Enterotoxin Genes. *Appl. Environ. Microbiol.* 76, 425–433. doi:10.1128/AEM.01357-09.

Kingombe, C. I., Huys, G., Tonolla, M., Albert, M. J., Swings, J., Peduzzi, R., et al. (1999). PCR detection, characterization, and distribution of virulence genes in *Aeromonas* spp. *Appl. Environ. Microbiol.* 65, 5293–5302.

Körkoca, H., Alan, Y., Bozari, S., Berktas, M., and Goz, Y. (2014). Detection of putative virulence genes in *Aeromonas* isolates from humans and animals. *J. Infect. Dev. Ctries.* 8, 1398–1406.

Králová, S., Staňková, E., and Sedláček, I. (2016). Classification of *Aeromonas* spp. isolated from water and clinical sources and distribution of virulence genes. *Folia Microbiol. (Praha)* 61, 513–521. doi:10.1007/s12223-016-0464-9.

Lago, E. P., Nieto, T. P., and Farto, R. (2012). Virulence factors of *Aeromonas salmonicida* subsp. *salmonicida* strains associated with infections in turbot *Psetta maxima*. *Dis. Aquat. Organ.* 99, 145–151. doi:10.3354/dao02467.

Li, F., Wang, W., Zhu, Z., Chen, A., Du, P., Wang, R., et al. (2015). Distribution, virulence-associated genes and antimicrobial resistance of *Aeromonas* isolates from diarrheal patients and water, China. *J. Infect.* 70, 600–608. doi:10.1016/j.jinf.2014.11.004.

Li, J., Ni, X. D., Liu, Y. J., and Lu, C. P. (2011). Detection of three virulence genes *alt*, *ahp* and *aerA* in *Aeromonas hydrophila* and their relationship with actual virulence to zebrafish. *J. Appl. Microbiol.* 110, 823–830. doi:10.1111/j.1365-2672.2011.04944.x.

Madetoja, J., Pylkkö, P., Pohjanvirta, T., Schildt, L., and Pelkonen, S. (2003). Putative virulence factors of atypical *Aeromonas salmonicida* isolated from Arctic charr, *Salvelinus alpinus* (L.), and European grayling, *Thymallus thymallus* (L.). *J. Fish Dis.* 26, 349–359.

Martínez, O., Rodríguez-Calleja, J. M., Santos, J. A., Otero, A., and García-López, M. L. (2009). Foodborne and indicator bacteria in farmed molluscan shellfish before and after depuration. *J. Food Prot.* 72, 1443–1449.

Martino, M. E., Fasolato, L., Montemurro, F., Rosteghin, M., Manfrin, A., Patarnello, T., et al. (2011). Determination of microbial diversity of *Aeromonas* strains on the basis of multilocus sequence typing, phenotype, and presence of putative virulence genes. *Appl. Environ. Microbiol.* 77, 4986–5000. doi:10.1128/AEM.00708-11.

Nagar, V., Shashidhar, R., and Bandekar, J. R. (2011). Prevalence, characterization, and antimicrobial resistance of *Aeromonas* strains from various retail food products in Mumbai, India. *J. Food Sci.* 76, M486-492. doi:10.1111/j.1750-3841.2011.02303.x.

Nam, I.-Y., and Joh, K. (2007). Rapid detection of virulence factors of *Aeromonas* isolated from a trout farm by hexaplex-PCR. *J. Microbiol. Seoul Korea* 45, 297–304.

Nawaz, M., Khan, S. A., Khan, A. A., Sung, K., Tran, Q., Kerdahi, K., et al. (2010). Detection and characterization of virulence genes and integrons in *Aeromonas veronii* isolated from catfish. *Food Microbiol.* 27, 327–331. doi:10.1016/j.fm.2009.11.007.

Nilsson, W. B., Gudkovs, N., and Strom, M. S. (2006). Atypical strains of *Aeromonas salmonicida* contain multiple copies of insertion element ISAsa4 useful as a genetic marker and a target for PCR assay. *Dis. Aquat. Organ.* 70, 209–217. doi:10.3354/dao070209.

Olaniran, A. O., Nzimande, S. B. T., and Mkize, N. G. (2015). Antimicrobial resistance and virulence signatures of *Listeria* and *Aeromonas* species recovered from treated wastewater effluent and receiving surface water in Durban, South Africa. *BMC Microbiol.* 15. doi:10.1186/s12866-015-0570-x.

Ørmen, Ø., Regue, M. Q., Tomás, J. M., and Granum, P. E. (2003). Studies of aerolysin promoters from different *Aeromonas* spp. *Microb. Pathog.* 35, 189–196.

Ottaviani, D., Parlani, C., Citterio, B., Masini, L., Leoni, F., Canonico, C., et al. (2011). Putative virulence properties of *Aeromonas* strains isolated from food, environmental and clinical sources in Italy: a comparative study. *Int. J. Food Microbiol.* 144, 538–545. doi:10.1016/j.ijfoodmicro.2010.11.020.

Pablos, M., Remacha, M.-A., Rodríguez-Calleja, J.-M., Santos, J. A., Otero, A., and García-López, M.-L. (2010). Identity, virulence genes, and clonal relatedness of *Aeromonas* isolates from patients with diarrhea and drinking water. *Eur. J. Clin. Microbiol. Infect. Dis. Off. Publ. Eur. Soc. Clin. Microbiol.* 29, 1163–1172. doi:10.1007/s10096-010-0982-3.

Pablos, M., Rodríguez-Calleja, J. M., Santos, J. A., Otero, A., and García-López, M.-L. (2009). Occurrence of motile *Aeromonas* in municipal drinking water and distribution of genes encoding virulence factors. *Int. J. Food Microbiol.* 135, 158–164. doi:10.1016/j.ijfoodmicro.2009.08.020.

Paton, A. W., and Paton, J. C. (1998). Detection and characterization of Shiga toxigenic *Escherichia coli* by using multiplex PCR assays for *stx1*, *stx2*, *eaeA*, enterohemorrhagic *E. coli hlyA*, *rfbO111*, and *rfbO157*. *J. Clin. Microbiol.* 36, 598–602.

Pillai, L., Sha, J., Erova, T. E., Fadl, A. A., Khajanchi, B. K., and Chopra, A. K. (2006). Molecular and functional characterization of a ToxR-regulated lipoprotein from a clinical isolate of *Aeromonas hydrophila*. *Infect. Immun.* 74, 3742–3755. doi:10.1128/IAI.00402-06.

Pollard, D. R., Johnson, W. M., Lior, H., Tyler, S. D., and Rozee, K. R. (1990). Detection of the aerolysin gene in *Aeromonas hydrophila* by the polymerase chain reaction. *J. Clin. Microbiol.* 28, 2477–2481.

Puthucheary, S. D., Puah, S. M., and Chua, K. H. (2012). Molecular characterization of clinical isolates of *Aeromonas species* from Malaysia. *PloS One* 7, e30205. doi:10.1371/journal.pone.0030205.

Robertson, B. K., Harden, C., Selvaraju, S. B., Pradhan, S., and Yadav, J. S. (2014). Molecular detection, quantification, and toxigenicity profiling of *Aeromonas* spp. in source- and drinking-water. *Open Microbiol. J.* 8, 32–39. doi:10.2174/1874285801408010032.

Santos, J. A., González, C. J., Otero, A., and García-López, M.-L. (1999). Hemolytic activity and siderophore production in different *Aeromonas* species isolated from fish. *Appl. Environ. Microbiol.* 65, 5612–5614.

Santos, P. G., Santos, P. A., Bello, A. R., and Freitas-Almeida, A. C. (2011). Association of *Aeromonas caviae* polar and lateral flagella with biofilm formation. *Lett. Appl. Microbiol.* 52, 49–55. doi:10.1111/j.1472-765X.2010.02965.x.

Sechi, L. A., Deriu, A., Falchi, M. P., Fadda, G., and Zanetti, S. (2002). Distribution of virulence genes in *Aeromonas* spp. isolated from Sardinian waters and from patients with diarrhoea. *J. Appl. Microbiol.* 92, 221–227.

Sen, K. (2005). Development of a rapid identification method for *Aeromonas* species by multiplex-PCR. *Can. J. Microbiol.* 51, 957–966. doi:10.1139/w05-089.

Sen, K., and Rodgers, M. (2004). Distribution of six virulence factors in *Aeromonas* species isolated from US drinking water utilities: a PCR identification. *J. Appl. Microbiol.* 97, 1077–1086. doi:10.1111/j.1365-2672.2004.02398.x.

Sha, J., Galindo, C. L., Pancholi, V., Popov, V. L., Zhao, Y., Houston, C. W., et al. (2003). Differential expression of the enolase gene under *in vivo* versus *in vitro* growth conditions of *Aeromonas hydrophila*. *Microb. Pathog.* 34, 195–204.

Sha, J., Kozlova, E. V., and Chopra, A. K. (2002). Role of various enterotoxins in *Aeromonas* hydrophila-induced gastroenteritis: generation of enterotoxin gene-deficient mutants and evaluation of their enterotoxic activity. *Infect. Immun.* 70, 1924–1935.

Sha, J., Kozlova, E. V., Fadl, A. A., Olano, J. P., Houston, C. W., Peterson, J. W., et al. (2004). Molecular characterization of a glucose-inhibited division gene, *gidA*, that regulates cytotoxic enterotoxin of *Aeromonas hydrophila*. *Infect. Immun.* 72, 1084–1095.

Sha, J., Pillai, L., Fadl, A. A., Galindo, C. L., Erova, T. E., and Chopra, A. K. (2005). The type III secretion system and cytotoxic enterotoxin alter the virulence of *Aeromonas hydrophila*. *Infect. Immun.* 73, 6446–6457. doi:10.1128/IAI.73.10.6446-6457.2005.

Sha, J., Wang, S. F., Suarez, G., Sierra, J. C., Fadl, A. A., Erova, T. E., et al. (2007). Further characterization of a type III secretion system (T3SS) and of a new effector protein from a clinical isolate of *Aeromonas hydrophila*--part I. *Microb. Pathog.* 43, 127–146. doi:10.1016/j.micpath.2007.05.002.

Silver, A. C., and Graf, J. (2009). Prevalence of genes encoding the type three secretion system and the effectors AexT and AexU in the *Aeromonas veronii* group. *DNA Cell Biol.* 28, 383–388. doi:10.1089/dna.2009.0867.

Sinha, S., Shimada, T., Ramamurthy, T., Bhattacharya, S. K., Yamasaki, S., Takeda, Y., et al. (2004). Prevalence, serotype distribution, antibiotic susceptibility and genetic profiles of mesophilic *Aeromonas* species isolated from hospitalized diarrhoeal cases in Kolkata, India. *J. Med. Microbiol.* 53, 527–534. doi:10.1099/jmm.0.05269-0.

Skwor, T., Shinko, J., Augustyniak, A., Gee, C., and Andraso, G. (2014). *Aeromonas hydrophila* and *Aeromonas veronii* predominate among potentially pathogenic ciprofloxacin- and tetracycline-resistant *Aeromonas* isolates from Lake Erie. *Appl. Environ. Microbiol.* 80, 841–848. doi:10.1128/AEM.03645-13.

Soler, L., Figueras, M. J., Chacón, M. R., Vila, J., Marco, F., Martinez-Murcia, A. J., et al. (2002). Potential virulence and antimicrobial susceptibility of *Aeromonas popoffii* recovered from freshwater and seawater. *FEMS Immunol. Med. Microbiol.* 32, 243–247.

Song, T., Toma, C., Nakasone, N., and Iwanaga, M. (2004). Aerolysin is activated by metalloprotease in *Aeromonas veronii* biovar *sobria*. *J. Med. Microbiol.* 53, 477–482. doi:10.1099/jmm.0.05405-0.

Suarez, G., Sierra, J. C., Sha, J., Wang, S., Erova, T. E., Fadl, A. A., et al. (2008). Molecular characterization of a functional type VI secretion system from a clinical isolate of *Aeromonas hydrophila*. *Microb. Pathog.* 44, 344–361. doi:10.1016/j.micpath.2007.10.005.

Vilches, S., Urgell, C., Merino, S., Chacón, M. R., Soler, L., Castro-Escarpulli, G., et al. (2004). Complete type III secretion system of a mesophilic *Aeromonas hydrophila* strain. *Appl. Environ. Microbiol.* 70, 6914–6919. doi:10.1128/AEM.70.11.6914-6919.2004.

Vilches, S., Wilhelms, M., Yu, H. B., Leung, K. Y., Tomás, J. M., and Merino, S. (2008). *Aeromonas hydrophila* AH-3 AexT is an ADP-ribosylating toxin secreted through the type III secretion system. *Microb. Pathog.* 44, 1–12. doi:10.1016/j.micpath.2007.06.004.

Wang, G., Clark, C. G., Liu, C., Pucknell, C., Munro, C. K., Kruk, T. M. A. C., et al. (2003). Detection and characterization of the hemolysin genes in *Aeromonas hydrophila* and *Aeromonas sobria* by multiplex PCR. *J. Clin. Microbiol.* 41, 1048–1054. doi:10.1128/JCM.41.3.1048-1054.2003.

Wang, G., Tyler, K. D., Munro, C. K., and Johnson, W. M. (1996). Characterization of cytotoxic, hemolytic *Aeromonas caviae* clinical isolates and their identification by determining presence of a unique hemolysin gene. *J. Clin. Microbiol.* 34, 3203–3205.

Wong, C. Y., Heuzenroeder, M. W., and Flower, R. L. (1998). Inactivation of two haemolytic toxin genes in *Aeromonas hydrophila* attenuates virulence in a suckling mouse model. *Microbiol. Read. Engl.* 144 ( Pt 2), 291–298. doi:10.1099/00221287-144-2-291.

Wu, C.-J., Wu, J.-J., Yan, J.-J., Lee, H.-C., Lee, N.-Y., Chang, C.-M., et al. (2007). Clinical significance and distribution of putative virulence markers of 116 consecutive clinical *Aeromonas* isolates in southern Taiwan. *J. Infect.* 54, 151–158. doi:10.1016/j.jinf.2006.04.002.

Yano, Y., Hamano, K., Tsutsui, I., Aue-Umneoy, D., Ban, M., and Satomi, M. (2015). Occurrence, molecular characterization, and antimicrobial susceptibility of *Aeromonas* spp. in marine species of shrimps cultured at inland low salinity ponds. *Food Microbiol.* 47, 21–27. doi:10.1016/j.fm.2014.11.003.

Ye, Y., Jiang, Y., Fan, T., Jiang, Q., Cheng, Y., Lu, J., et al. (2012). Resistance characterization, virulence factors, and ERIC-PCR fingerprinting of *Aeromonas veronii* strains isolated from diseased Trionyx sinensis. *Foodborne Pathog. Dis.* 9, 1053–1055. doi:10.1089/fpd.2012.1181.

Yi, S.-W., You, M.-J., Cho, H.-S., Lee, C.-S., Kwon, J.-K., and Shin, G.-W. (2013). Molecular characterization of *Aeromonas* species isolated from farmed eels (*Anguilla japonica*). *Vet. Microbiol.* 164, 195–200. doi:10.1016/j.vetmic.2013.02.006.

Yu, C.-P., and Chu, K.-H. (2011). Molecular quantification of virulence gene-containing *Aeromonas* in water samples collected from different drinking water treatment processes. *Environ. Monit. Assess.* 176, 225–238. doi:10.1007/s10661-010-1578-1.

Yu, C.-P., Farrell, S. K., Robinson, B., and Chu, K.-H. (2008). Development and application of real-time PCR assays for quantifying total and aerolysin gene-containing *Aeromonas* in source, intermediate, and finished drinking water. *Environ. Sci. Technol.* 42, 1191–1200.
