# Supplementary material for: Genome-driven evaluation and redesign of PCR tools for improving the detection of virulence-associated genes in aeromonads
Source: PLoS One. 2018 Aug 15;13(8):e0201428. doi: 10.1371/journal.pone.0201428 (PMC6093642; doi:10.1371/journal.pone.0201428)
Supplement: S2 Table — (DOCX) [file pone.0201428.s004.docx]

**S2 Table. Additional nucleic sequence accession numbers used to design new primers.**

| Gene | Additional nucleic sequences used to design new primers |
| --- | --- |
| *aer/act* | GB M16495 (aerolysin/preprotoxin, *A. bestiarum* Ah65 {*A. hydrophila* Ah65}†)  EMBL X65044 (hemolysin/aerolysin, *A. hydrophila* 28SA)  EMBL X65045 (hemolysin/aerolysin, *A. hydrophila* AH1)  GB M84709 (cytolytic enterotoxin, *A. dhakensis* SSU {*A. hydrophila*})  GB U40711 (hemolysin, *A. caviae* A1833)  GB AF064068 (preproaerolysin, *A. trota* ATCC 49659)  EMBL Y00559 (aerolysin, *A. sobria* AB3)  EMBL X65046 (hemolysin/aerolysin, *A. sobria* 33)  EMBL X65048 (beta-hemolysin, *A. salmonicida* 17-2) |
| *ser* | EMBL X67043 (aspA serine protease, *A.salmonicida* MT0004-U2862)  GB AF159142 (ahe2 serine protease, *A. hydrophila* AG2)  EMBL ABO90569 (ahe2 serine protease, *A. salmonicida* subsp. *salmonicida* A449) EMBL AEB49525 (ahe2 serine protease, *A. veronii* B565)  EMBL EQC03180 ahe2 serine protease, *A. salmonicida* subsp. *pectinolytica* 34mel)  EMBL AHE49971 (ahe2 serine protease, *A. rivipollensis* 4AK4 {*A. hydrophila* 4AK4})  EMBL AY841795 (serA serine protease, *A. hydrophila* AH1)  EMBL EKB19572 (microbial serine protease, *A. veronii* AMC34)  EMBL AAZ67922 (serine protease, *A. hydrophila* PHP99)  EMBL EKB28603 (serine protease, *A. dhakensis* SSU {*A. hydrophila* SSU})  EMBL ACV96792 (serine protease, *A. hydrophila* BSK-10)  EMBL EKB21457 (serine protease, *A. veronii* AMC35) |
| *asc-FG* | EMBL AY289195 (AscF and AscG, *A. veronii* 283c)  EMBL AJ616218 (AscF and AscG, *A. salmonicida subsp. salmonicida* JF2267)  EMBL HM584607 (AscF and AscG, *A. veronii* bv. *veronii* AMC35)  EMBL HM584606 (AscF and AscG, *A. veronii* bv. *veronii* AMC34)  EMBL HM584590 (AscF and AscG, *A. veronii* bv. *sobria* LMG 13659)  EMBL HM584589 (AscF and AscG, *A. allosaccharophila* LMG 140549)  EMBL JN676202 (AscF and AscG, *Aeromonas* sp. MCCB 140)  EMBL JN676201 (AscF and AscG, *Aeromonas* sp. MCCB 142)  EMBL JN676200 (AscF and AscG, *Aeromonas* sp. MTHA) |
| *asc-V* | EMBL CAD30218 (AscV, *A. salmonicida* subsp. *salmonicida* JF2267)  EMBL ABP51929 (AscV, *A. veronii* bv. *sobria* HM21)  EMBL AAV30230 (AscV, *A. dhakensis* SSU {*A. hydrophila* SSU})  EMBL CAG44555 (AscV, *A. sobria* JF2635)  EMBL AAR26336 (AscV, *A. hydrophila* AH-1)  EMBL EHI50383 (AscV, *A. salmonicida* subsp. *salmonicida* 01-B526)  EMBL ENY73366 (AscV, *A. diversa* 2478-85)  EMBL ABO92544 (AscV, *A. salmonicida* subsp. *salmonicida* A449)  EMBL AAS91816 (AscV, *A. piscicola* AH-3 {*A. hydrophila* AH-3}) |
| *lafA* | EMBL AAK57644 (LafA1, *A. caviae* Sch3 {*A. punctate* Sch3})  EMBL AAK57645 (LafA2, *A. caviae* Sch3 {*A. punctate* Sch3})  EMBL AAK20919 (lateral flagellin LafA, *A. piscicola* AH-3 {*A. hydrophila* AH-3})  EMBL ABG56545 (LafA1, *A. hydrophila* AH-1)  EMBL ABG56546 (LafA2, *A. hydrophila* AH-1)  EMBL AAP51733 (partial lateral flagellin LafA, *A. jandaei* A919)  EMBL AHE51206 (LafA1, *A. rivipollensis* 4AK4 {*A. hydrophila* 4AK4})  EMBL AHE51207 (LafA2, *A. rivipollensis* 4AK4 {*A. hydrophila* 4AK4}) |

†Previously published names are indicated inside braces.
